# Thermodynamic Characterization of the Ca^2+^-Dependent Interaction Between SOUL and ALG-2

**DOI:** 10.3390/ijms19123802

**Published:** 2018-11-29

**Authors:** Taisuke Mikasa, Masami Kugo, Seigo Nishimura, Sigeru Taketani, Sumio Ishijima, Ikuko Sagami

**Affiliations:** 1 Department of Applied Life Science, Graduate School of Life and Environmental Science, Kyoto Prefectural University, Sakyouku Shimogamo Nakaragi 1-5, Kyoto 606-8522, Japan; hbbjf981@yahoo.co.jp (T.M.); gonkugo@gmail.com (M.K.); say_go_happy@yahoo.co.jp (S.N.); ishijima@kpu.ac.jp (S.I.); 2 Department of Biotechnology, Kyoto Institute of Technology, Sakyo-ku, Kyoto 606-8585, Japan; taketani.shigeru.5a@kyoto-u.ac.jp

**Keywords:** ALG-2, calcium-binding protein, SOUL, HEBP-2, heme-binding protein, thermodynamics, ITC, binding affinity

## Abstract

SOUL, a heme-binding protein-2 (HEBP-2), interacts with apoptosis-linked gene 2 protein (ALG-2) in a Ca^2+^-dependent manner. To investigate the properties of the interaction of SOUL with ALG-2, we generated several mutants of SOUL and ALG-2 and analyzed the recombinant proteins using pulldown assay and isothermal titration calorimetry. The interaction between SOUL and ALG-2 (delta3-23ALG-2) was an exothermic reaction, with 1:1 stoichiometry and high affinity (*Kd* = 32.4 nM) in the presence of Ca^2+^. The heat capacity change (Δ*Cp*) of the reaction showed a large negative value (−390 cal/K·mol), which suggested the burial of a significant nonpolar surface area or disruption of a hydrogen bond network that was induced by the interaction (or both). One-point mutation of SOUL Phe100 or ALG-2 Trp57 resulted in complete loss of heat change, supporting the essential roles of these residues for the interaction. Nevertheless, a truncated mutant of SOUL1-143 that deleted the domain required for the interaction with ALG-2 Trp57 still showed 1:1 binding to ALG-2 with an endothermic reaction. These results provide a better understanding of the target recognition mechanism and conformational change of SOUL in the interaction with ALG-2.

## 1. Introduction

**Figure 1:**
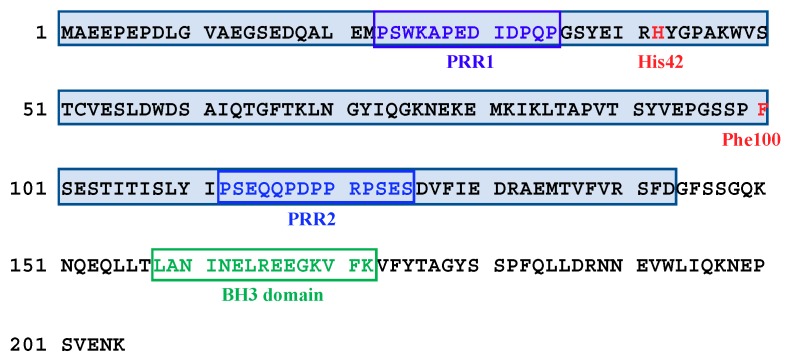
Amino acid sequences of mice SOUL. Blue letters: Pro-rich region 1 (PRR1) and Pro-rich region 2 (PRR2). Green letters: BH3 domain [9,10]. His42 is a heme-binding site [7]. Red F is Phe100. Pale blue shadow: the region of 1–143 amino acids.

SOUL (also known as heme-binding protein 2, HEBP2) was initially identified as a protein associated with the circadian rhythm or light perception in the retina, and the pineal gland of chickens [1]. Subsequently, the gene coding for this protein was found in various species, including *Arabidopsis thaliana*, mice, and humans [2]. Despite the ubiquitous presence across the tree of life, the functional role of SOUL in cells still remains poorly understood. In *Arabidopsis*, SOUL orthologs are implicated in phytochrome-mediated red or far-red light responses [3], and in the heme oxygenase-mediated antioxidant pathway [4]. In mice, p22HBP (HEBP1), which exhibits more than 40% sequence homology with SOUL, was characterized as a cytosolic, heme-binding protein [5] responsible for hemoglobin biosynthesis as a heme transporter or chaperone [6]. The mouse SOUL (mSOUL) protein also bound to heme with His42 as an endogenous ligand; it is a dimer in the apo-form that hexamerizes upon binding to heme [7]. Although these biochemical characterizations of SOUL suggest its roles in the regulation of porphyrin pool and heme transport in both plants and animals, the roles of heme-binding to SOUL are not sufficiently clear. In addition, the role of mammalian SOUL in cell death has been suggested, because mammalian SOUL contains a Bcl-2-homology 3 (BH3) sequence, which is a conserved domain in the apoptotic Bcl-2 protein family (Figure 1) [8,9]. Indeed, a synthetic oligopeptide of the BH3 domain of SOUL interacted with the anti-apoptotic protein Bcl-xL [10]. However, the precise molecular roles of SOUL in the cell remain unclear.

Recently, SOUL was reported as a protein that interacts with ALG-2 (Apoptosis-Linked Gene 2, also known as PDCD6) according to the studies of proteome-scale maps of the human protein–protein interaction [11,12] and a yeast two-hybrid screen with ALG-2 as the bait [13]. We have also independently identified ALG-2 by screening a mouse brain cDNA library using a yeast two-hybrid system with SOUL as the bait. ALG-2 was originally isolated in a screen for genes associated with T-cell receptor-mediated apoptosis [14]. Although apoptosis was not blocked in mice that are deficient for ALG-2 [15], accumulating evidence has revealed various roles of ALG-2 in the cell. ALG-2 is a penta-EF hand protein and interacts in a Ca^2+^-dependent manner with various proteins that are linked to several important cellular activities, including ER-to-Golgi trafficking and endosomal sorting/transport (ESCRT) [16,17]. Three kinds of proline-rich region (PRR) in target proteins are reported as ALG-2 binding motifs (ABMs). ABM-1 (Type-1 motif) contains a consensus sequence PPYPXXPGYP (X represents any residue) that is found in ALIX and TSG101 of ESCRT complexes, and PLSCR3 involved in cardiolipin translocation. ABM-2 (Type-2 motif) is found in SEC31 of the coat protein complex II (COPII) and PLSCR3, and contains a PXPGF sequence [16,17]. Some proteins, such as PLSCR3, contain both ABM-1 and ABM-2 motifs. Third, ABM-3 (Type-3 motif), which is found in the ESCRT-III related protein IST1, has a MPMPMPMP sequence [18]. ALG-2 can also bind with proteins that contain no obvious PRRs, such as Mucolipin-1 that has an ABH (acidic/basic/hydrophobic cluster) motif [19]. In addition, there are two ALG-2 isoforms, wild-type ALG-2 (ALG-2^WT^) and a splice variant ALG-2^ΔGF^, which contains a deletion of Gly121 and Phe122 in mammalian cells, at a ratio of 2:1 in mice [20] and 3:1 in humans [16]. While ALG-2^WT^ can interact with each of the ABM motifs in the target proteins, ALG-2^ΔGF^ interacts with ABM-2, but not with ABM-1 and ABM-3 [20,21,22], which indicates that the deletion of GF effects target-protein specificity.

SOUL contains two Pro-rich regions that do not share any apparent consensus sequence with ABM-1, ABM-2, and ABM-3 (Figure 1). We generated various recombinant truncated mutants of SOUL in an *E. coli* expression system and analyzed its interaction with ALG-2 in vitro using pulldown assays to elucidate the ALG-2 binding region of SOUL. Recently, during our experiments, the crystal structure of the ALG-2/HEBP2 (SOUL) complex was obtained, which demonstrated that ALG-2 interacted with the region of SOUL that was completely different from that of a canonical ABM [13]. Our results were consistent with those obtained from the crystal structure. Then, to further characterize the binding of SOUL to ALG-2 in solution, we examined the thermodynamic profiles associated with formation of the SOUL/ALG-2 complex using isothermal titration calorimetry (ITC). ITC is a useful technique that is capable of quantifying the stoichiometry, equilibrium constants, and thermodynamics of binding. To the best of our knowledge, this is the first study to demonstrate the thermodynamic parameters, such as the dissociation constant (*Kd*), Gibbs free energy (Δ*G*), enthalpy (Δ*H*), and entropy (TΔ*S*), for the interaction between Ca^2+^-bound ALG-2 and the full-length target protein. We also analyzed interactions using SOUL mutants or ALG-2 mutants including ALG-2^ΔGF^, and we discuss the thermodynamics.

## 2. Results

### 2.1. Ca^2+^-Dependent Interaction Between SOUL^WT^ and ALG-2^WT^

**Figure 2:**
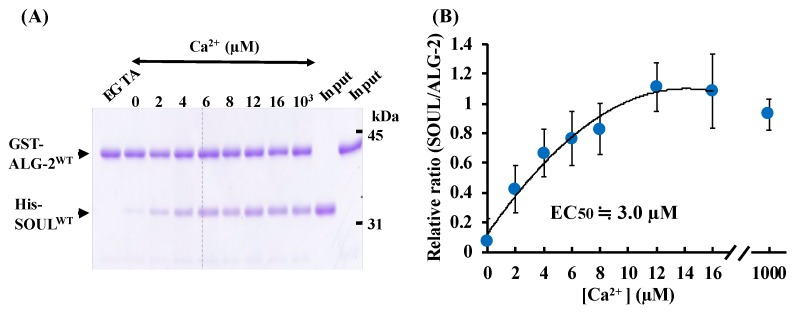
Effects of Ca^2+^ or EGTA on the interaction between SOUL and ALG-2. (**A**) GST-pulldown assays of GST-ALG-2^WT^ with His-SOUL^WT^ in the presence of various amounts of Ca^2+^ or 1 mM EGTA in vitro. (**B**) Relationship between [Ca^2+^] and the binding activity. The activity is represented as a relative value of the SOUL/ALG-2 quantified by Multi Gauge version 2.1. Each value is the mean of at least three independent experiments ± SD.

We generated wild-type SOUL fused to His-tag (His-SOUL^WT^) and wild-type ALG-2 fused to GST-tag (GST-ALG-2^WT^) using an *E. coli* expression system, and purified these proteins and performed GST-tag pulldown assays in vitro in the presence of EGTA (ethylene glycol teraacetic acid) or various amounts of Ca^2+^ (Figure 2). SOUL^WT^ interacted with ALG-2^WT^ in a Ca^2+^-dependent manner. However, the addition of EGTA to the reaction mixture abrogated the interaction in these pulldown experiments. A half-maximal effective concentration (EC50) was 3.0 µM of Ca^2+^. Pulldown analysis using His-tag of His-SOUL gave similar results.

**Figure 3:**
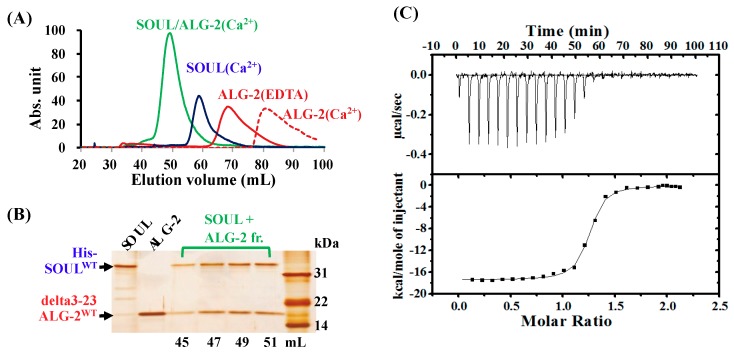
Interaction between His-SOUL^WT^ and delta2-23 ALG-2^WT^. (**A**) Tetrameric complex formation of His-SOUL^WT^ with delta2-23 ALG-2^WT^ in solution. Each protein sample (5 μM) was analyzed on a Sephacryl-S-200 HR column. Elution profiles of 5 µM of His-SOUL^WT^ (blue line), 5 µM of delta3-23ALG-2^WT^ (dotted red line), the mixture (green line) of 5 µM of each protein in the presence of 20 µM Ca^2+^, or delta3-23ALG-2^WT^ in the presence of 2 mM EDTA (red line). (**B**) Sliver staining after SDS-PAGE of fractions eluted from the gel-filtration of the mixture. (**C**) ITC measurements for the interactions between SOUL^WT^ and delta3-23 ALG-2^WT^. Typical calorimetric titrations (upper panel) and the resulting integrated binding isotherm (lower panel) at 25 °C and pH 7.5 in the presence of 15 µM Ca^2+^. 5 μL aliquots of 130 µM His-SOUL^WT^ were injected into 5 µM delta3-23ALG2^WT^. After subtracting the heat of ligand dilution, the solid line connecting the integrated data points was obtained from a one-set-of-sites model fitting using a nonlinear least-squares method.

To further characterize the interaction of SOUL^WT^ with ALG-2^WT^, we used isothermal titration calorimetry (ITC) in solution. Because of the low solubility of full-length ALG-2^WT^ without GST-tag in the buffer containing Ca^2+^ [23], we used an N-terminal truncated protein (delta3-23ALG-2^WT^) lacking the hydrophobic 21 residues in the presence of 0.25% (*v*/*v*) Tween 20 for ITC measurements. Purified delta3-23ALG-2^WT^ interacted with His-SOUL^WT^ similarly to full-length GST-ALG-2^WT^ in His-tag pulldown assay. The interaction was also confirmed by gel-filtration analysis (Figure 3A,B). His-SOUL^WT^ and the mixture with delta3-23ALG-2^WT^ were run on a size exclusion column under identical buffer conditions containing 20 µM CaCl_2_. Delta3-23ALG-2^WT^ was also analyzed in a buffer containing 20 µM CaCl_2_ or 2 mM EDTA. Comparison of the elution volumes with those obtained for proteins with known molecular weights indicated that His-SOUL^WT^ alone was a dimer, as described previously (Figure S1) [7]. Delta3-23ALG-2^WT^ was eluted as a monomer in the presence of EDTA, but at the fractions with much smaller molecular weight than monomer size in the presence of Ca^2+^, probably due to a non-specific association to the column resin. These are consistent with the previous report that ALG-2 dose not form a stable dimer without target protein in solution [16]. However, chemical-cross linking experiments and analytical ultracentrifugation analysis suggest that ALG-2 exists predominantly as a dimer, and the crystal structures of ALG-2 were solved as dimers [16]. On the other hand, when equal amounts of His-SOUL^WT^ and delta3-23ALG-2^WT^ were mixed in the presence of Ca^2+^ before gel-filtration, the proteins were mostly eluted as the tetramer complex. Two protein bands of SOUL^WT^ and delta3-23ALG-2^WT^ were clearly observed in the eluted fractions after SDS-PAGE (Figure 3B), which confirmed the complex formation. These data indicate that SOUL and delta3-23ALG-2 form a tetramer with 1:1 stoichiometry in the presence of Ca^2+^. We show, for the first time to our knowledge, the tetramer formation of the ALG-2 dimer with two molecules of the full-length target protein in solution.

Next, delta3-23ALG-2^WT^ was subjected to titrations with Ca^2+^ (Figure S2). The ITC measurements were performed at 25 °C in 0.15 M KCl with 25 mM HEPES, 0.25% Tween 20, and 10% glycerol at pH 7.5; these conditions were similar to those employed in ^45^Ca^2+^ flow-dialysis studies on the full-length proteins [20,24] and in an ITC study on the des23ALG-2^WT^, which has a deletion of N-terminal 23 amino acids [25], except that 0.50% Tween 20 was used in the previous studies. Under our experimental conditions, delta3-23ALG-2^WT^ contains two high affinity sites (*Kd*; 0.25 and 3.1 µM) and one low affinity site (*Kd*; 410 µM) of Ca^2+^, which is consistent with earlier works [20,24,25]. Even with deleting the N-terminal, delta3-23ALG-2^WT^ with too much Ca^2+^ showed a tendency to aggregate during long-term ITC experiments. Therefore, we conducted titrations of SOUL^WT^ into delta3-23ALG-2^WT^ in the presence of 15 µM Ca^2+^.

Figure 3C demonstrates a typical binding isotherm and plotted titration curve for the binding of His-SOUL^WT^ to delta3-23ALG-2^WT^ in the presence of 15 µM Ca^2+^ at 25 °C, pH 7.5. The exothermic reaction was observed, and after subtracting dilution heat, the obtained heat data were described well by a one-set-of-sites model. Consistent with the gel filtration results, the ITC data demonstrated that the calculated binding stoichiometry (n) was 1, which indicated that a single SOUL molecule binds to each Ca^2+^-bound ALG-2 molecule. The thermodynamic parameters obtained from at least three independent ITC experiments were *Kd* = 32.4 ± 6.6 nM, ∆*H* = −17.9 ± 0.7 kcal/mol, ∆*G* = −10.2 ± 0.2 kcal/mol, and T∆*S* = −7.6 ± 0.6 kcal/mol, as summarized in Table 1. We also analyzed the data with a sequential-binding-sites model; each of two SOUL molecules sequentially and cooperatively might bind to two sites on ALG-2 molecules during tetramer complex formation. However, one ITC datum gave a similar *Kd* value for each of the first and second bindings of SOUL to ALG-2 (37 and 40 nM, respectively) at half saturation of each ALG-2 site, while another did not give reliable results. On the other hand, analysis using a one-set-of-sites model gave us a good fit to the experimental data and highly reliable results. These results indicated no obvious cooperativity in the interaction between SOUL and ALG-2. Therefore, we used a one-set-of-sites model for the other ITC analyses.

**Figure 4:**
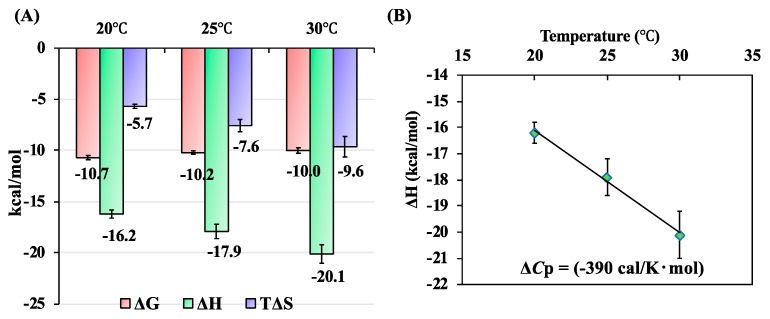
(**A**) The temperature dependence of the thermodynamic parameters in binding of SOUL^WT^ to delta3-23ALG-2^WT^. The Gibbs free energy change for binding (Δ*G*) is represented in red, the enthalpy change for binding (Δ*H*) is represented in green, and the entropy change for binding (TΔ*S*) is represented in purple. ITC measurements were performed in triplicate at each temperature. The observed Δ*H* values were plotted versus temperature (**B**), and the Δ*Cp* value was obtained from its slope.

To evaluate the temperature dependence of the thermodynamic parameters in the binding of SOUL to ALG-2, the calorimetric titrations were performed at 20, 25, and 30 °C (Figure 4). The Δ*H* values for the binding decreased with temperature, and this was accompanied by a decrease in the TΔ*S* values. As a result, the Δ*G* values for the binding were insensitive to temperature. The heat capacity change (Δ*Cp*) of the binding reaction, as estimated from the slope of the plot of Δ*H* versus temperature, was large and of negative value (−390 cal·mol^−1^·K^−1^).

### 2.2. Interaction of SOUL^WT^ with the ALG-2^∆GF^ Variant

**Figure 5:**
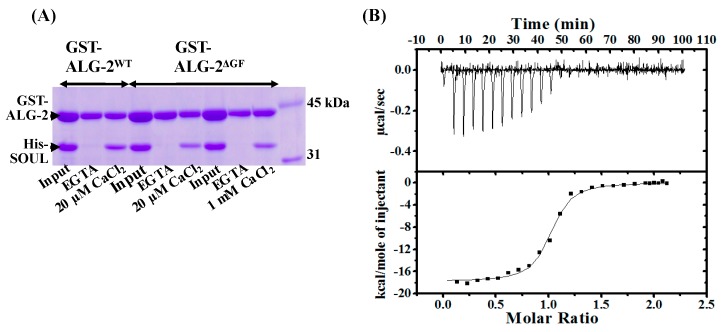
Interaction between SOUL^WT^ and ALG-2^∆GF^. (**A**) GST-pulldown assays using His-SOUL (5 µM) and GST-ALG-2^∆GF^ (5 µM) proteins under the same conditions as described in Figure 3C. (**B**) The typical isothermal titration calorimetric profiles of the interaction between His-SOUL^WT^ and delta3-23ALG-2^∆GF^ in the presence of 15 µM Ca^2+^ at 25 °C and pH 7.5.

An isoform of ALG-2^∆GF^, with deletion of Gly121 and Phe122, was first reported as a shorter cDNA clone in six nucleotides corresponding to the two amino acids in comparison with the full-length cDNA clone of ALG-2^WT^ in mammalian cells [22]. Interestingly, ALG-2^∆GF^ interacts exclusively with ABM-2 in the target proteins, while ALG-2^WT^ can interact with either ABM-1 or -2. In this study, we generated GST-ALG-2^∆GF^ for GST-pulldown assay, and delta3-23ALG-2^∆GF^ for ITC experiments, to analyze their interaction with SOUL^WT^. Similar to ALG-2^WT^, ALG-2^∆GF^ interacted with SOUL^WT^ in the presence of 20 µM and 1 mM Ca^2+^, equally (Figure 5A). Figure 5B shows a typical ITC profile of SOUL^WT^ titration into delta3-23ALG-2^∆GF^, and the thermodynamic parameters are summarized in comparison with those to ALG-2^WT^ in Table 1. The interaction between SOUL^WT^ and delta3-23ALG-2^∆GF^ showed an exothermic reaction. The ∆*H* value (−19.8 kcal/mol) of SOUL^WT^ binding to delta3-23ALG-2^∆GF^ was more favorable, but the T∆*S* value (−9.4 kcal/mol) was increased. As a result, the binding affinity was slightly lower than that of the wild type, but still quite high (*Kd* = 62.0 nM).

### 2.3. Effects of the Mutation of SOUL Phe100 and ALG-2 Trp57

**Figure 6:**
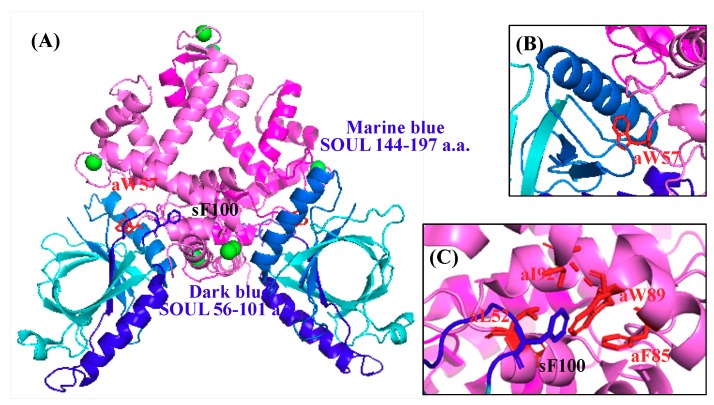
Structure of the human SOUL/ALG-2 complex. (**A**) Location of SOUL56-101, Phe100 of SOUL and Trp57 of ALG-2 in the structure of the tetrameric SOUL/ALG-2 complex (PDB code: 5gqq) [13]. Blue, SOUL; dark blue, SOUL56-101 amino acids; marine blue, SOUL144-197 amino acids that were deleted in a SOUL1-143 mutant; sF100, SOUL Phe100; pink, ALG-2; aW57, ALG-2 Trp57; green boles, Ca^2+^ ions. (**B**) Interaction sites of ALG-2 Trp57 with the SOUL C-terminal region. (**C**) Interaction sites of SOUL Phe100 in a hydrophobic pocket (pocket 3) of ALG-2.

Resent structural analysis of the human SOUL/ALG-2 complex revealed that SOUL Phe100 and ALG-2 Trp57 could be anchors to the respective hydrophobic pocket of the counterpart, as shown in Figure 6 [13]. In their pulldown analysis, a single mutation in either SOUL or ALG-2 resulted in a significant decrease of the interaction, but still retained some complex formation.

**Figure 7:**
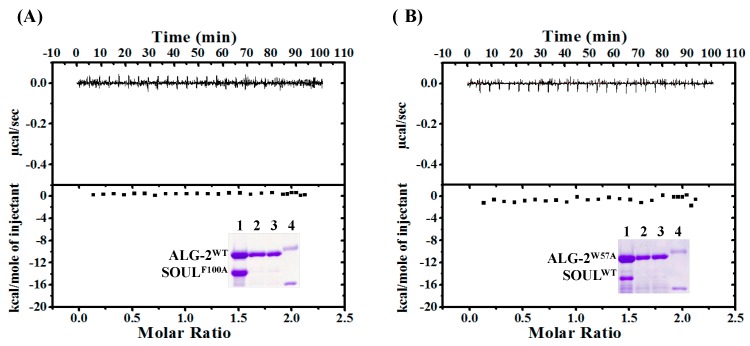
Calorimetric titrations and GST-pulldown analysis using SOUL^F100A^ or ALG-2^W57A^. Titrations of His-SOUL^F100A^ to delta3-23ALG-2^WT^ (**A**) or His-SOUL^WT^ to delta3-23ALG-2^W57A^ (**B**) were performed under the same conditions as described in Figure 3C. Insets show GST-pulldown analysis of His-SOUL^F100A^ and GST-ALG-2^WT^ (**A**) or His-SOUL^WT^ and GST-ALG-2^W57A^ (**B**) in the presence of 20 µM Ca^2+^. Lane 1, input; lane 2, + 1 mM EGTA; lane 3, + 20 µM Ca^2+^; Lane 4, protein markers, 45 and 31 kDa.

To evaluate the effects of one-point-mutation of these residues on the interaction, we generated His-SOUL^F100A^ and GST-ALG-2^W57A^ for GST-pulldown assay, and delta3-23ALG-2 ^W57A^ for ITC measurement. Under our experimental conditions, either interaction between SOUL^F100A^ and ALG-2^WT^ or between SOUL^WT^ and ALG-2^W57A^ was not observed, even in GST-pulldown analysis (Figure 7, insets). In addition, calorimetric titrations of SOUL^F100A^ to delta3-23ALG-2^WT^ and SOUL^WT^ to delta3-23ALG-2 ^W57A^ exhibited no heat change (Figure 7). These results demonstrated that mutation of SOUL Phe100 or ALG-2 Trp57 resulted in complete loss of complex formation.

### 2.4. Interactions of the Truncated SOUL Mutants and ALG-2^WT^

**Figure 8:**
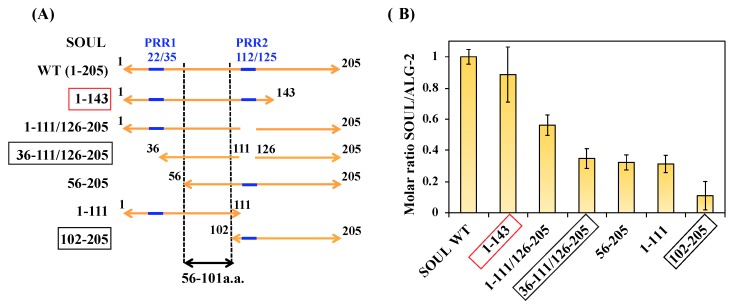
Interactions of SOUL mutants with ALG-2 in vitro. (**A**) PRR1 is 23-PSWKAPEDIDPQP-35 and PRR2 is 112-PSEQQPDPPRPSES-125 in blue. (**B**) GST-pulldown assays were performed using various purified mutants of His-SOUL (5 µM) and purified GST-ALG-2^WT^ (5 µM) proteins in the presence of Ca^2+^ or EGTA. The intensity of the bands stained after SDS-PAGE was quantified by Multi Gauge version 2.1, and is represented as a relative value normalized to the value obtained with His-SOUL^WT^ and GST-ALG-2^WT^ in the presence of 20 µM Ca^2+^. Each value is the mean of at least three independent experiments ± SD.

In the structure of the SOUL/ALG-2 complex, Trp57 of ALG-2 is located within a hydrophobic pocket formed with the C-terminal half of SOUL, while Phe100 of SOUL bound to the near pocket 3 formed by residues from EF1 to EF3 of ALG-2 (Figure 6) [13]. The region of SOUL containing Phe100 is not proline-rich and not also similar to a non-Pro-based motif found in the proteins that are known to interact with ALG-2 [16,17]. Considering these reports, it is interesting to know the minimum domain of SOUL required for interaction with ALG-2. We generated several truncated mutants of SOUL fused to His-tag and performed GST-pulldown assay with GST-ALG-2^WT^ (Figure S3). We found two proline-rich regions (PRR1 and 2) without similarity to any ABM in the primary sequence of SOUL (Figure 1). PRR1 is 22-PSWKAPEDIDPQP-35 and PRR2 is 112-PSEQQPDPPRPSES-125. As shown in Figure 8, deletion of either PRR1(56-205) or PRR2 (1-111/126-205) of SOUL reduced the interaction to 32% and 56% in comparison with SOUL^WT^. A SOUL mutant (36-111/126-205) without two PRRs and a SOUL1-111 lacking the C-terminal half still retained the capability to interact with ALG-2. In contrast, SOUL102-205, with a deletion of the N-terminal half, mostly abolished the binding with ALG-2. Taken together, these results indicated that the region containing 56-101 residues of SOUL was important for interaction with ALG-2.

**Figure 9:**
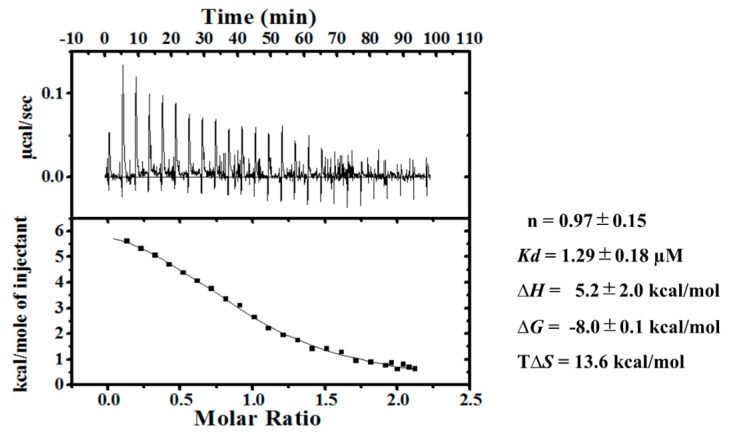
ITC measurements for the interactions between SOUL1-143 and delta3-23 ALG-2^WT^. Typical calorimetric titrations (upper panel) and the resulting integrated binding isotherm (lower panel) at 25 °C and pH 7.5 in the presence of 15 µM Ca^2+^. The thermodynamic parameters were obtained from the analysis of three-independent ITC data.

Among the SOUL mutants, SOUL1-143 significantly bound to ALG-2 (about 90% in comparison with SOUL^WT^), although the deleted region forms an interacting domain for ALG-2 Typ57, as shown in Figure 6 [13]. For further evaluation of the interaction, we performed ITC experiments using His-SOUL1-143 and delta3-23ALG-2^WT^ (Figure 9). Notably, the interaction was endothermic and resulted in a positive enthalpy change (Δ*H*), in contrast to the interaction of SOUL^WT^. This unfavorable contribution to the binding free energy (Δ*G*) was overcome by the positive net entropy change (TΔ*S*). As a result, SOUL1-143 bound to ALG-2^WT^ with a 1:1 stoichiometry, but with a low affinity (*Kd*; 1.3 µM). As expected, the CD spectrum of SOUL1-143 was different from that of SOUL^WT^, with a decrease in alpha-helix content (22% of SOUL^WT^, 14% of SOUL1-143) [26,27] (Figure S4), which indicated the deletion of the alpha-helix formed by the C-terminal.

## 3. Discussion

In mammals, SOUL is expressed ubiquitously in various tissues, and the level has been found to be especially high in pancreas adenocarcinoma and in some cancer-derived cell lines, including Panc-1, Jurkat, and HeLa cells [9]. It’s overexpression can facilitate permeabilization of both outer and inner mitochondrial membranes, and cell death that is induced by calcium ionophore. These suggest the possible involvement of SOUL in calcium signaling [8]. However, little information is available to verify the direct commitment of calcium for the function of SOUL. In this study, we found that SOUL interacted with ALG-2 in a Ca^2+^-dependent manner and formed a tetramer complex in a buffer containing a physiological concentration of Ca^2+^. These results are consistent with the data that has been recently reported by Ma J. et. al [13]. They identified SOUL as an interacting partner for ALG-2 through a yeast two-hybrid screening, and solved the crystal structure of the ALG-2/SOUL tetramer complex.

ALG-2 is a 22 kDa Ca^2+^-binding protein of the penta-EF-hand protein family, which includes sorcin, grancalcin, and calpains, and interacts with various proteins in a Ca^2+^-dependent manner [16,17]. Ca^2+^ binding induced a conformational change in ALG-2 that exposed its hydrophobic surface; subsequently, ALG-2 interacted with the target proteins [23]. Previous flow-dialysis studies on the full-length proteins [20,24], and an ITC study on the des23ALG-2^WT^ [25] in the presence of 0.5% Tween 20, revealed two high affinity sites and one lower affinity site for Ca^2+^ binding. Within five repeats of the EF-hand motif in ALG-2, EF-1 and EF-3 have been identified as the high affinity sites for Ca^2+^ binding [23,24] that exhibited a typical coordination with Ca^2+^ in the crystal structure [25,28,29]. EF5 had an incomplete Ca^2+^ coordination, which indicated a low affinity site. Deletion of the N-terminal of ALG-2^WT^ to increase protein solubilization had little effect on the Ca^2+^ affinity and Ca^2+^-induced conformational change [20,24]. Consistent with these results, our ITC data demonstrated that delta3-23ALG-2^WT^ showed two high affinity sites and one lower affinity site for Ca^2+^ binding under our experimental conditions in the presence of 0.25% Tween 20 (Figure S2). Each *Kd* value was of a similar order to those previously reported. GST-pulldown assay data (EC50; 3.0 µM) also supported that high affinity sites being occupied with Ca^2+^ were enough to induce a conformational change of GST-ALG-2^WT^ for interaction with His-SOUL^WT^ (Figure 2).

Our ITC data revealed that full-length SOUL^WT^ interacted with Ca^2+^-bound delta3-23ALG-2 with a 1:1 binding stoichiometry, without cooperativity and with a *Kd* value of 32.4 nM. As shown in Table 2, this nM order affinity of SOUL is significantly higher than that of ALIX peptide [24] and Sorcin peptide (µM order) [30], and is comparable with that of PLSCR peptides [21] and Annexin peptides [31], for binding to ALG-2.

These suggest that SOUL can preferentially associate with ALG-2, even when ALIX and Sorcin (or both) coexist in the cells. The enthalpy change (Δ*H*) of SOUL binding was a large negative (–17.9 kcal/mol), indicating multiple favorable noncovalent interactions between two molecules, even though it was accompanied by a negative enthalpy change (TΔ*S*) as common enthalpy–entropy compensation [32]. The Δ*G* values for the binding were insensitive to temperature, due to the compensatory effects of enthalpy and entropy, which are temperature dependent (Figure 4). The heat capacity change (Δ*Cp*) was a large negative, which suggested burial of the hydrophobic surface area during the specific interaction [32,33]. In addition, water molecule networks in the interface were also suggested to contribute to a negative Δ*Cp* in protein–ligand interactions [32,34,35]. A recent crystal structure of the SOUL/ALG-2 complex revealed that the interacting interface between SOUL and ALG-2 is significantly larger in comparison with that between ALIX peptide and ALG-2 [13]. The calculation of buried surface areas by using each crystal structure revealed that the buried area of the SOUL/ALG-2 tetramer complex (9120 Å^2^) is much larger than that (5820 Å^2^) calculated from each structure of SOUL dimers and ALG-2 dimers. The ALG-2 dimer in the SOUL/ALG-2 complex showed a more compact conformation as compared with the dimer structure of free ALG-2 or the complex with the ALIX peptide. These structural data support the high binding affinity and large negative Δ*Cp* value of SOUL, which has been demonstrated in our study.

ALG-2 has a natural splice variant ALG-2^ΔGF^, which lacks residues Gly121 and Phe122 located in EF3. ALG-2^ΔGF^ cannot interact with ALIX and TSG101 containing the binding motif ABM-1, which occupies the hydrophobic pockets 1 and 2 on ALG-2 in the peptide/ALG-2 complex [20,21,22]. On the other hand, SEC31A and PLSCR3 containing ABM-2 can bind not only ALG-2^WT^ but also ALG-2^ΔGF^ [21], and SEC31A peptide occupies pocket 3 on ALG-2 in the crystal structure [36]. Pockets 1 and 2 are largely formed by residues from EF3 to EF5, while pocket 3 is formed by residues from EF1 to EF3 [17,36]. The crystal structure of the SOUL/ALG-2 tetramer complex revealed that the interaction interface between SOUL and ALG-2 overlaps partially with pocket 3 found in the SEC31/ALG-2 complex [13,34]. Our ITC experiments demonstrated that the binding affinity of SOUL to ALG-2^ΔGF^ was lower but comparable to that to ALG-2^WT^ (Figure 5). These results indicated that the deletion of two residues in ALG-2 did not noticeably affect the thermodynamics of SOUL binding. In pocket 3 of ALG-2, a single mutation of Leu52 or Phe85 to Ala caused loss of interaction with SEC31 [34]. We also generated several mutants in pocket 3 and investigated the contributions of these residues to the interaction with SOUL via a GST-pulldown assay. Binding of L52A or F85A to SOUL was reduced but was still 80~90% of ALG-2^WT^, while W89A and I92A showed no change in interactions (Figure S5). These results again suggest that the SOUL binding site on ALG-2 is near pocket 3, and the binding mode is similar but not identical to that of SEC31. We also tried ITC experiments of these mutants, but could not get good results, because delta3-23ALG-2 mutants were more easy to aggregate in ITC buffer than the wild-type.

Pulldown analysis using the truncated mutants of SOUL demonstrated that the 56-101 region from the N-terminal of SOUL is essential for the interaction with ALG-2. While the mutant 102-205 with deletion of Phe100 hardly interacted with ALG-2, all mutants of SOUL that interacted with ALG-2 contain Phe100. These results are consistent with structural analysis that showed that Phe100 of SOUL is suggested to be an anchor residue for the interaction in the SOUL/ALG-2 complex (Figure 6) [13]. The structure of SOUL/ALG-2 also revealed that Trp57 of ALG-2 could be an anchor for SOUL. Under our conditions, either single mutation of F100A or W57A completely disrupted the complex formation, as demonstrated in GST-pulldown assay and in ITC experiments (Figure 7). These data indicate that both Phe100 of SOUL and Trp57 of ALG-2 are essential for the interaction between two proteins. In the structure of the SOUL/ALG-2 complex, the C-terminal half of SOUL seems to interact with Trp57 of ALG-2 (Figure 6) [13]. Nevertheless, our pulldown analysis (Figure 8) and ITC analysis (Figure 9) indicated that SOUL1-143 lacking the C-terminal half significantly interacted with ALG-2. The thermodynamics revealed that the interaction was endothermic, but showed a large positive enthalpy change (TΔ*S*). These results suggest that Trp57 of ALG-2 interacted with a different hydrophobic pocket in SOUL1-143 in a different fashion. Such flexible recognition by ALG-2 might be a reason for its binding ability with a wide variety of target proteins.

In summary, SOUL interacts with a hydrophobic pocket near pocket 3 on ALG-2 in a Ca^2+^-dependent manner. ITC results indicated that the interaction is exothermic and the binding affinity is in the nM order, supporting the physiological relevance of SOUL to Ca^2+^ signaling through interaction with ALG-2 in cells. To date, we have no information about post translational modification for SOUL, except heme binding [7,8]. Studies of heme-bound SOUL are now in progress to investigate the role of heme in SOUL function. In addition, future research on the structures and thermodynamics of full-length target proteins with Ca^2+^-bound ALG-2 will help to understand the diverse and selective recognition mechanisms of ALG-2, and its roles as a mediator for Ca^2+^-signaling in cells.

## 4. Materials and Methods

### 4.1. Plasmid Construction

To construct the expression plasmids for full-length murine SOUL^WT^ (1–205 amino acids) with His-tag and murine ALG-2^WT^ (1–191 amino acids), and glutathione-S-transferase (GST)-tag, the corresponding cDNAs were cloned into the *Nde* I and *Sal* I sites of pET28a(+) (Novagen, Madison, WI, USA) (*Soul*/pET28) and the *BamH* I and *Sal* I sites of pGEX-4T-2 (GE Healthcare Japan) (*Alg-2*/pGEX), respectively.

Another plasmid, delta3-23Alg-2/pET-11a was constructed into the *Nde* I and *BamH* I sites of pET-11a to obtain a soluble ALG-2 protein (24–191 amino acids) without tag for the ITC experiment. To generate various mutants of SOUL or ALG-2, PCR-based mutagenesis was performed using appropriate primer sets (Figure S6), and the desired mutation was confirmed via sequencing.

### 4.2. Preparation of SOUL and ALG-2 Proteins

Expression and purification of His-tagged SOULs were performed basically, as previously described [7], except that *E. coli* BL21(DE3), which contained another plasmid (pGroESL, a gift from DuPont, Wilmington, DE, USA) for the expression of chaperone proteins, was used as the host cell. Wild-type SOUL or the F100A mutant of SOUL was eluted from a Ni-NTA-agarose column with buffer A (50 mM Na-phosphate (pH 7.5), 1 mM PMSF, 2 μg/mL aprotinin, 2 μg/mL leupeptin, 2 μg/mL pepstatin, 1 mM DTT) containing 100 mM imidazole, after washing the column with buffer A containing 50 mM imidazole. The other mutants were eluted with buffer A containing 500 mM imidazole, after washing with buffer A containing 100 mM imidazole. The protein was passed through a Sephadex G-25 column pre-equilibrated with buffer B (100 mM Tris-HCl (pH 7.5), 100 mM NaCl, and 10% (*v*/*v*) glycerol) or buffer C (25 mM HEPES (pH 7.5), 150 mM NaCl, and 10% (*v*/*v*) glycerol).

GST-tagged ALG-2 was expressed in *E. coli* BL21-CodonPlus(DE3)-(RIPL) harboring an expression vector (*Alg-2*/pGEX) in TB medium. Protein expression was induced by the addition of a final concentration of 0.10 mM IPTG at OD600nm = 0.6, and the cells were further incubated at 15 °C for 20 h after addition of IPTG. *E. coli* cells were crushed in buffer D (1 × PBS (pH 7.2), 1 mM PMSF, 2 μg/mL aprotinin, 2 μg/mL leupeptin, 2 μg/mL pepstatin, 1 mM DTT) by pulsed sonication. After ultracentrifugation, ammonium sulfate was added to the resulting supernatant up to 60% saturation. Precipitates were collected and dissolved in buffer D. The solution was passed through a Sephadex G-25 column (4 × 20 cm) pre-equilibrated with the same buffer. The eluted solution was applied to a Glutathione Sepharose column pre-equilibrated with buffer D. After washing the column with buffer D, ALG-2 protein was eluted with buffer E (50 mM Tris-HCl (pH 8.0), 10 mM reduced glutathione, and 10% (*v*/*v*) glycerol). The protein was passed through a Sephadex G-25 column pre-equilibrated with buffer B or buffer C to exchange the buffer for future experiments.

Delta3-23ALG-2, a protein with deletion of the N-terminal 3–23 amino acids was used for gel filtration and isothermal titration calorimetry (ITC) experiments, because full-length ALG-2 without tag was easy to aggregate in the absence of Ca^2+^ [22]. Delta3-23ALG-2 was expressed in *E. coli* BL21-CodonPlus(DE3)-(RIPL) harboring an expression vector (delta3-23*Alg-2*/pGEX) in TB medium. Protein expression was performed as described for GST-ALG-2. Purification of delta3-23ALG-2 was conducted basically, as previously described [37]. Briefly, *E. coli* cells were crushed in buffer F (50 mM Tris-HCl (pH 7.5), 2 mM EDTA, 1 mM PMSF, 2 μg/mL aprotinin, 2 μg/mL leupeptin, 2 μg/mL pepstatin, and 0.2 mM DTT) by pulsed sonication. After ultracentrifugation, CaCl_2_ solution (final 3 mM) was added to the supernatant and the mixture was incubated at 4 °C for 10 min. The precipitates were collected and dissolved in buffer F via sonication (repeated twice). After centrifugation at 10,000× *g* for 30 min, the supernatant was applied to a HiTrap Q column pre-equilibrated with 50 mM Tris-HCl (pH 7.5), and delta3-23 ALG-2 was eluted using a gradient elution with 50 mM Tris-HCl (pH 7.5) containing 0.4 M NaCl. The delta3-23 ALG-2 protein fractions were collected and incubated at 4 °C for 1 h after addition of the final 1mM EDTA to remove metal ions. The protein was then further purified using a Sephacryl S-200 HR column pre-equilibrated with buffer B or buffer C.

The concentration of each purified protein was determined by Bradford protein assay, and by SDS-PAGE using bovine serum albumin as a standard.

### 4.3. Pulldown Assay

Interaction between SOUL and ALG-2 was examined by pulldown assay. Purified His-SOUL (5 µM) and GST-ALG-2 (5 µM) were mixed with 50 μL of Glutathione Sepharose 4B (GE Healthcare Japan) in 200 µL of buffer B, containing 20 µM CaCl_2_ or 1 mM EGTA, and incubated with gentle tapping for 30 min at 4 °C. After two washes with buffer B containing 20 µM CaCl_2_ or 1 mM EGTA, the bound proteins were eluted from the resin with 50 mM Tris-HCl (pH 8.0) containing 20 mM reduced glutathione, and 20 µM CaCl_2_ or 1 mM EGTA, respectively. The proteins in the eluates were separated by SDS-PAGE and detected by Coomassie brilliant blue staining. Pulldown assays using Ni-NTA agarose resin were also performed for His-SOUL. The bound proteins were eluted with 250 mM imidazole in buffer B containing CaCl_2_ or EGTA.

### 4.4. Gel filtration Analysis

The mobility of His-SOUL^WT^, delta3-23 ALG-2^WT^, or the mixture of His-SOUL^WT^/delta3-23 ALG-2^WT^ was analyzed using a gel filtration assay. Briefly, 1 mL of each protein sample (5 μM) was run on a Sephacryl-S-200 HR (150 mL) column (GE Healthcare) in buffer B containing 20 µM CaCl_2_ or 2 mM EDTA. Protein fractions eluted were analyzed by SDS-PAGE. A low molecular weight calibration kit (GE Healthcare) was used to determine the molecular weight of the complexes.

### 4.5. Isothermal Titration Calorimetry (ITC) Measurements

ITC experiments were performed for thermodynamic analyses of the interactions between His-SOUL and delta3-23ALG-2 proteins using an MCS-ITC (MicroCal Inc., Northampton, MA, USA). Before titration of delta3-23ALG-2 with SOUL, delta3-23ALG-2 was titrated with Ca^2+^ at 25 °C in 25 mM HEPES (pH 7.5), 150 mM NaCl, and 10% (*v*/*v*) glycerol (buffer C). Purified delta3-23ALG-2 after removal of metal ions by EDTA treatment was dialyzed against buffer C and filtrated. Next, final 0.25% (*v*/*v*) Tween 20 was added to the sample to prevent protein precipitation during the experiment. Delta3-23ALG-2 (10 µM) was titrated with 5-µl of 440 µM CaCl_2_ after an initial 2-µl injection. Baseline correction was performed by subtracting the heat of dilution measured by titration of CaCl_2_ into the buffer. Binding curves were analyzed, and dissociation constants (*Kd*) were determined by a “three-set-of-sites” model using MicroCal Origin 5.0 software supplied by the manufacturer (MicroCal Inc., Northampton, MA, USA).

ITC measurements for His-SOUL and delta3-23ALG-2 were conducted in buffer C that contained 15 µM CaCl_2_ and 0.25% (*v*/*v*) Tween 20. Delta3-23ALG-2 (5 or 10 µM) was titrated with SOUL (130 µM or 260 µM). The data were collected for a total of 25 injections at 4 min intervals. The first injection included only 2 μL of ligand, and the corresponding data point was deleted from the analysis. The heat for each injection was subtracted from the heat of dilution of the injectant, which was measured by injecting SOUL solution into the experimental buffer. Each corrected heat was divided by the molar concentration of SOUL injected, and was analyzed on the basis of a “one-set-of sites” model or a “sequential-binding-sites” model using MicroCal Origin 5.0 software. Finally, the reported values were obtained from the analysis using a “one-set-of sites” fitting model and an average of at least three ITC runs. The binding stoichiometry (n), the association constant (*Ka*), and the enthalpy change (Δ*H*) were obtained from the fitted curve. The values of the Gibbs free energy change (Δ*G*) and the entropy change (Δ) were calculated from the following equation:

Δ*G* = −RtlnKa = ΔH − TΔ (1)

where *R* is the gas constant and T is the absolute temperature. The dissociation constant *Kd* is also used in the text (*Kd* = 1/*Ka*). The Δ*Cp* was calculated from the slope of the regression line of linear fit of the ΔH values that were measured at three different temperatures—20, 25, and 30 °C.

### 4.6. Circular Dichroism (CD) Spectra

To examine the secondary structure of the protein sample, CD spectra were obtained at 20 °C in buffer C using a Jasco J-720 CD spectrometer. To ensure that the temperature of the solution was appropriate, a purified protein sample (5 µM) was incubated for 10 min prior to spectroscopic measurements.

## Figures and Tables

**Table 1:** Thermodynamic parameters for the interaction between SOUL^WT^ and delta3-23ALG-2^WT^ or delta3-23ALG-2^∆GF^ at 25 °C.

ALG-2 Proteins	*n*	*Kd* (nM)	Δ*H* (kcal/mol)	Δ*G* (kcal/mol)	T∆*S* (kcal/mol)
delta3-23ALG-2^WT^	1.16 ± 0.02	32.4 ± 6.6	−17.9 ± 0.7	−10.2 ± 0.2	−7.6 ± 0.6
delta3-23ALG-2^∆GF^	1.00 ± 0.07	62.0 ± 13.7	−19.8 ± 2.0	−9.8 ± 0.1	−9.4 ± 1.6

**Table 2:** Binding affinity of the target protein or peptide with Ca^2+^/ALG-2.

Target Protein (Peptide)	Binding Affinity (*Kd*)	Method	Reference
SOUL	32.4 nM	ITC	this work
ALIX (799-814: QGPPYPTYPGYPGYSQ) *	~0.2 µM	SPR	[24]
Annexin A7 (4-PGYPPPPGGYP) *	60 nM, 0.7 µM	SPR	[31]
Annexin A11 (4-PGYPPPPGGYP)) *	40 nM, 0.5 µM	SPR	[31]
PLSCR3 ABS-1 peptide (KGYAPSPPPPYPVTPGYPEPA) * ABS-2 peptide (KQVPAPAPGFALFPSPGPVA) *	40 nM 25 nM	SPR SPR	[21]
Sorcin (12-GYYPGG)	5 µM	SPR	[30]

* underline, amino acid residues compatible with ALG-2 binding motifs (ABMs).

## Supplementary Materials

Supplementary materials can be found at http://www.mdpi.com/1422-0067/19/12/3802/s1.

## Abbreviations

ABM	ALG-2-binding motif
ALIX	ALG-2-interacting protein X
Δ*Cp*	Heat capacity change
ESCRT	Endosomal sorting complex required for transport
HEBP	Heme-binding protein
PAGE	Polyacrylamide gel electrophoresis
PLSCR3	Phospholipid scramblase 3
PRR	Proline-rich region
TSG101	Tumor susceptibility gene 101
