# Supplementary material for: Thermodynamic Characterization of the Ca2+-Dependent Interaction Between SOUL and ALG-2
Source: Int J Mol Sci. 2018 Nov 29;19(12):3802. doi: 10.3390/ijms19123802 (PMC6321638; doi:10.3390/ijms19123802)
Supplement: Supplementary file 1 [file ijms-19-03802-s001.pdf]

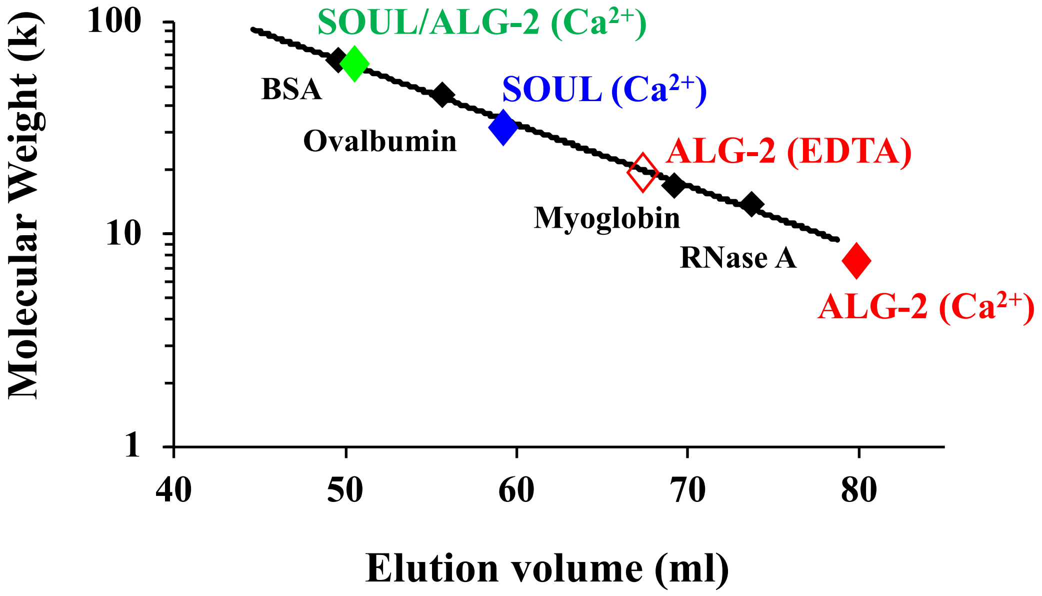

Figure S1. Correlation between the elution volumes of gel filtration and molecular weights of various proteins. His-SOUL^{WT}/delta2-23ALG-2^{WT} complex (filled green), SOUL^{WT} (filled blue), delta3-23ALG-2^{WT} (filled red) in the presence of Ca^{2+}, and delta2-23ALG-2^{WT} (open red) in the presence of EDTA.

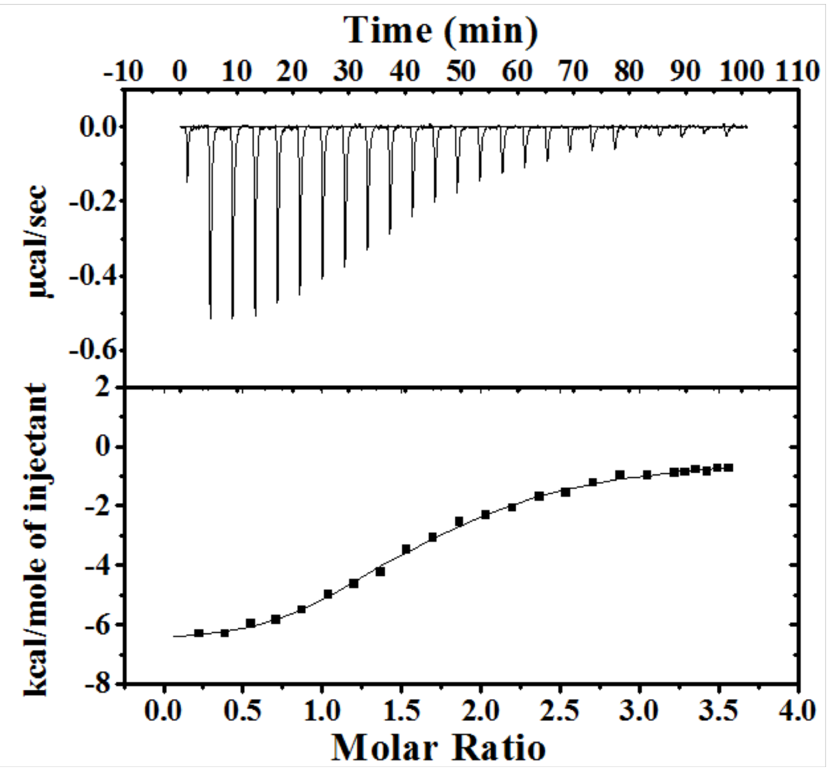

Figure S2. Determination of Ca^{2+}-binding parameters by ITC.
Typical calorimetric titrations (A) and the resulting integrated binding isotherm (B) at 25°C, pH 7.5 in buffer C. After subtracting the heat of ligand dilution, the solid line connecting the integrated data points was obtained from a three-set-of-sites model fitting using a nonlinear least-squares method.

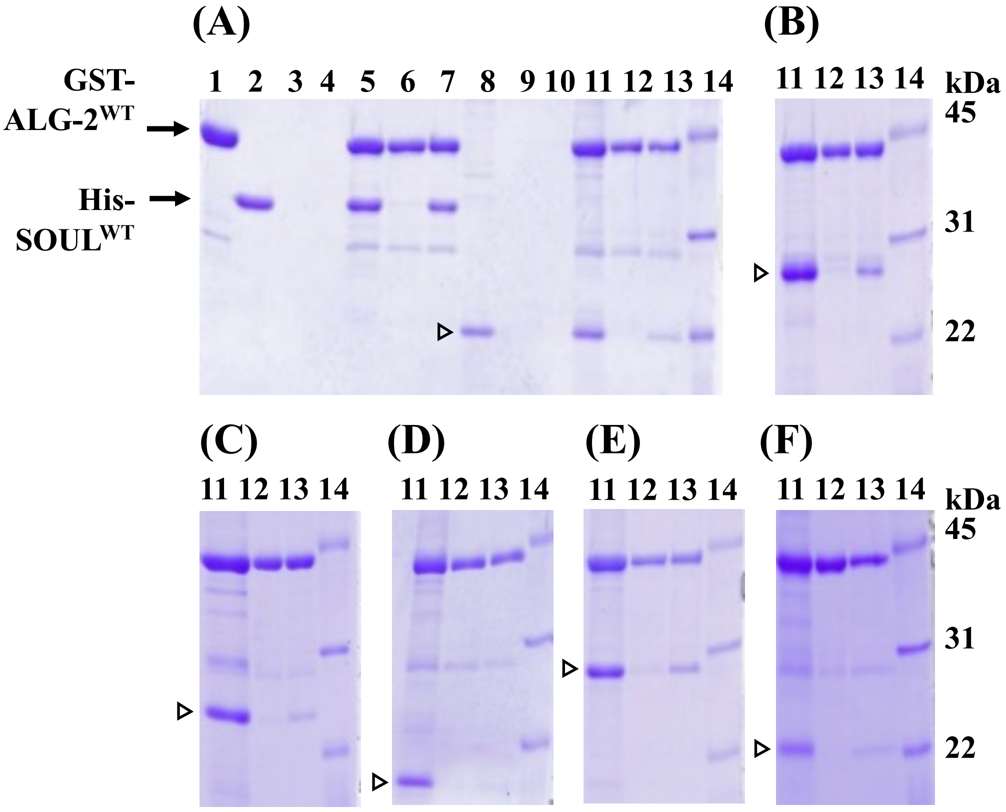

Figure S3. GST-pulldown assays using SOUL mutants.
GST-ALG-2^{WT} protein was incubated with His-SOUL wild-type(WT), 1-111 (A), 1-143 (B), 56-205 (C), 102-205 (D), 1-111/126-205 (E), and 36-111/126-205 (F).
The bound proteins were analyzed in 12.5%SDS-PAGE.
1: GST-ALG-2^{WT} input, 2: His-$SOUL^{WT}$ input, 3:His-$SOUL^{WT}$ with 1 mM EGTA eluate, 4: His-$SOUL^{WT}$ with 20 μM $CaCl_2$ eluate, 5: GST-ALG-2^{WT}+ His-$SOUL^{WT}$ input, 6: GST-ALG-2^{WT}+ His-$SOUL^{WT}$ with 1 mM EGTA, 7: GST-ALG-2^{WT}+ His-$SOUL^{WT}$ with 20 μM $CaCl_2$, 8: His-SOUL mutant input, 9: His-SOUL mutant with 1 mM EGTA , 10: His-SOUL mutant with 20 μM $CaCl_2$, 11: GST-ALG-2^{WT} + His-SOUL mutant input, 12: GST-ALG-2^{WT} + His-SOUL mutant with 1 mM EGTA, 13: GST-ALG-2^{WT} + His-SOUL mutant with 20 μM $CaCl_2$, 14: protein marker. Each triangle indicates each mutant.

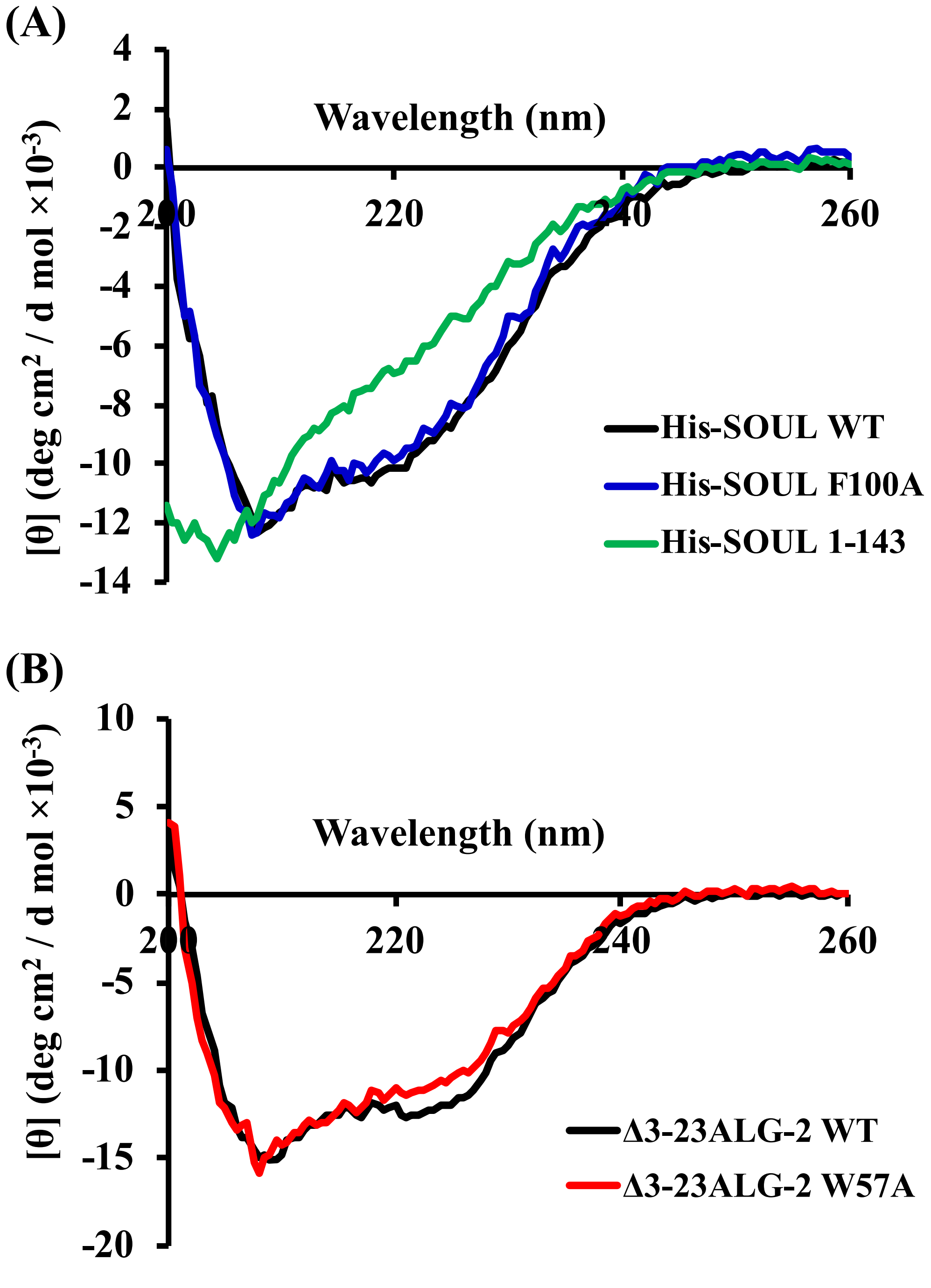

Figure S4. Circular dichroism (CD) spectra of SOULs (A) and ALG-2s (B). CD spectra were obtained at 20°C from 260 to 200 nm. Scanning rate was set to 50 nm/min. Loading concentrations of proteins were 5 μM in a buffer B. The background signal from the buffer solution was subtracted from each spectrum.

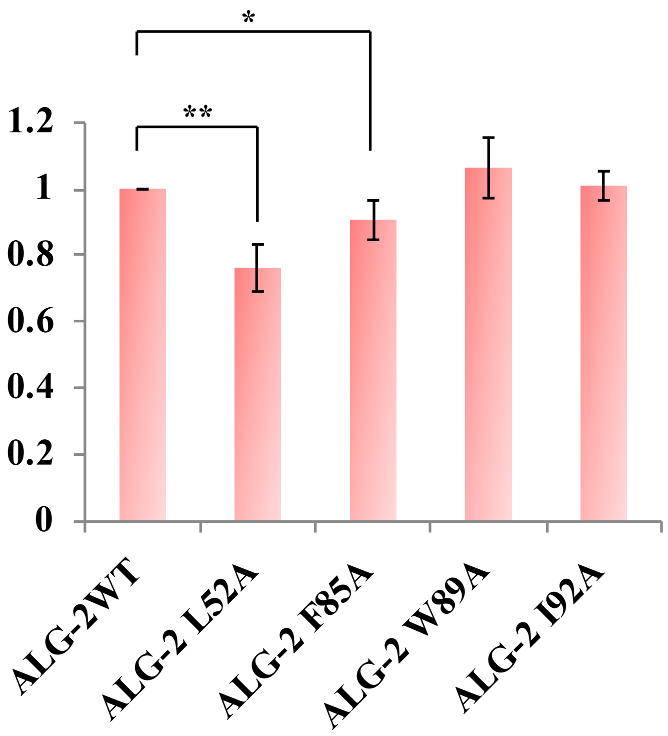

Figure S5. Interaction between GST-ALG-2 mutant and His-SOUL^{WT}. GST-pulldown assays were performed using various purified mutants of GST-ALG-2 (5 μM) and purified His-SOUL^{WT} (5 μM) proteins in the presence of 20 μM Ca^{2+}. The intensity of the bands stained after SDS-PAGE was quantified by Multi Gauge version 2.1 and is represented as a relative value normalized to the value obtained with His-SOUL^{WT} and GST-ALG-2^{WT}. Each value is the mean of at least three independent experiments ± SD. *$P < 0.05$, **$P < 0.01$

mSOUL Primers

N2s	: 5’-CGGGATCCCATATGGCAGAGGAGCC-3’
205Stopa	: 5’-GCAAGCTTATTTGTTCTCGACGGAGG-3’
143Stopa	: 5’-GCAAGCTTAATCAAAAGACCGCACGAACAC-3’
111Cla1a	: 5’-GGATCCATCGATGTACAGGGAAATCGTAATGGTAG-3’
126Cla1s	: 5’-CATATGATCGATGTCTTCATTGAAGACAGAGCTG-3’
36s	: 5’-CGGGATCCCATATGGGAAGTTATGAGATCC-3’
56s	: 5’-GGGATCCCATATGCTGGACTGGGATTCAGC-3’
111Stopa	: 5’-GCAAGCTTAGATGTACAGGGAAATCGTAATGG-3’
102a	: 5’-GGGATCCCATATGGAGTCTACCATTACG-5’
F100A	
Forward primer	: 5’-CCGGCTCAAGTCCTGCGAGTGAGTCTACCATTACG-3’
Reverse primer	: 5’-CGTAATGGTAGACTCACTCGCAGGACTTGAGCCGG-3’

ALG-2 Primers

Delat3-23	
Forward primer	: 5’- CGGGATCCCATATGGCTGACCAGAGCTTCCTGTGG-3’
Reverse primer	: 5’- GCGGATCCTTATACAATGCTGAAGACCATGGAGAG-3’
L52A	
Forward primer	: 5’- GAGCTTCAGCAAGCAGCGTCCAATGGTACATGGAC-3’
Reverse primer	: 5’- GTCCATGTACCATTGGACGCTGCTTGCTGAAGCTC-3’
W57A	
Forward primer	: 5’- GCATTATCCAATGGTACAGCGACTCCATTTAACCC -3’
Reverse primer	: 5’- GGGTTAAATGGAGTCGCTGTACCATTGGATAATGC-3’
F85A	
Forward primer	: 5’- GTGTGAACTTCAGTGAAGCGACGGGTGTGTGGAAG-3’
Reverse primer	: 5’- CTTCCACACACCCGTCGCTTCACTGAAGTTCACAC -3’
W89A	
Forward primer	: 5’- CAGTGAATTCACGGGTGTGGCGAAGTATATCACAG-3’
Reverse primer	: 5’- CTGTGATATACTTCGCCACACCCGTGAATTCACTG-3’
I92A	
Forward primer	: 5’- GGGTGTGTGGAAGTATGCGACAGACTGGCAGAATG -3’
Reverse primer	: 5’- CATTCTGCCAGTCTGTCGCATACTTCCACACACCC-3’

Figure S6. Primers for plasmid construction
